# Cardioprotective Effects of QiShenYiQi Dripping Pills on Transverse Aortic Constriction-Induced Heart Failure in Mice

**DOI:** 10.3389/fphys.2018.00324

**Published:** 2018-04-03

**Authors:** Guoran Ruan, Haojin Ren, Chi Zhang, Xiaogang Zhu, Chao Xu, Liyue Wang

**Affiliations:** Department of Cardiology, The Puren Hospital, Wuhan University of Science and Technology, Wuhan, China

**Keywords:** traditional Chinese medicine, QiShenYiQi dripping pills, heart failure, cardiac remodeling, apoptosis, fibrosis, angiogenesis

## Abstract

QiShenYiQi dripping pills (QSYQ), a traditional Chinese medicine, are commonly used to treat coronary heart disease, and QSYQ was recently approved as a complementary treatment for ischemic heart failure in China. However, only few studies reported on whether QSYQ exerts a protective effect on heart failure induced by pressure overload. In this study, we explored the role of QSYQ in a mouse model of heart failure induced by transverse aortic constriction (TAC). Twenty-eight C57BL/6J mice were divided into four groups: Sham + NS group, Sham + QSYQ group, TAC + NS group, and TAC + QSYQ group. QSYQ dissolved in normal saline (NS) was administered intragastrically (3.5 mg/100 g/day) in the Sham + QSYQ and TAC + QSYQ groups. In the Sham + NS and TAC + NS groups, NS was provided every day intragastrically. Eight weeks after TAC, echocardiography, and cardiac catheterization were performed to evaluate the cardiac function, and immunofluorescent staining with anti-actinin2 antibody was performed to determine the structure of the myocardial fibers. Moreover, TUNEL staining and Masson trichrome staining were employed to assess the effects of QSYQ on cardiac apoptosis and cardiac fibrosis. Western blots and real-time polymerase chain reaction (PCR) were used to measure the expression levels of vascular endothelial growth factor (VEGF) in the heart, and immunohistochemical staining with anti-CD31 antibody was performed to explore the role of QSYQ in cardiac angiogenesis. Results showed that TAC-induced cardiac dysfunction and disrupted structure of myocardial fibers significantly improved after QSYQ treatment. Moreover, QSYQ treatment also significantly improved cardiac apoptosis and cardiac fibrosis in TAC-induced heart failure, which was accompanied by an increase in VEGF expression levels and maintenance of microvessel density in the heart. In conclusion, QSYQ exerts a protective effect on TAC-induced heart failure, which could be attributed to enhanced cardiac angiogenesis, which is closely related to QSYQ. Thus, QSYQ may be a promising traditional Chinese medicine for the treatment of heart failure induced by pressure overload such as hypertension.

## Introduction

QiShenYiQi dripping pills (QSYQ) is a compound Chinese medicine, which is composed of six herbs including 2 star herbs: Astragalus membranaceus (Fisch.) Bunge (“huang-qi” in Chinese) and Salvia Miltiorrhiza Bunge (“danshen” in Chinese), and 4 adjunctive herbs: Lonicera japonica Thunb., Scrophularia aestivalis Griseb., Aconitum fischeri Rchb., and Glycyrrhiza uralensis Fisch (Wang et al., 2017). QSYQ is approved by the China State Food and Drug Administration in 2003 for the treatment of coronary heart disease and angina pectoris (Wang et al., 2011). Recently, QSYQ was used as a complementary treatment for ischemic heart failure in China (Hou et al., 2013; Wang et al., 2013). However, only few studies that explore the potential effect of QSYQ on pressure overload-induced heart failure have been conducted. Although some studies reported that QSYQ could attenuate pressure overload-induced cardiac hypertrophy and myocardial fibrosis (Li et al., 2012; Chen et al., 2015; Lv et al., 2015), studies using various methodologies to draw a more convincing conclusion on the potential effect of QSYQ on pressure overload-induced heart failure remain essential.

Heart failure is the ultimate result of a large number of cardiovascular diseases, which are a leading causes of morbidity and mortality worldwide (Shah and Mann, 2011). It is regarded as a progressive and irreversible process characterized by cardiac pump failure and cardiac remodeling (Hou and Kang, 2012). Pathological cardiac remodeling, resulting from cardiac pressure, volume overload, or ischemic injury, is considered the most crucial mechanism for the development of heart failure (Ho et al., 2010; Ahmad et al., 2012) and is a progressive and irreversible process characterized by cardiac hypertrophy, cardiac apoptosis, and cardiac fibrosis (Cohn et al., 2000). Therefore, preventing or reversing pathological cardiac remodeling is an effective way to prevent heart failure (Kirkpatrick and St John Sutton, 2012).

Angiogenesis plays a critical protective role in pressure-overload heart failure by preventing cardiac remodeling (Shiojima et al., 2005; Oka et al., 2014). Myocardial angiogenesis is regulated by secreted angiogenic growth factors, including VEGFs, fibroblast growth factors (Kardami et al., 2007), angiopoietin-1and-2 (Dallabrida et al., 2008), platelet-derived growth factors (Andrae et al., 2008), and transforming growth factors (Dobaczewski et al., 2011). Among them, VEGFs are one of prime regulators of myocardial angiogenesis. Overexpression of VEGF improves heart failure by maintaining myocardial capillary density while downregulation of VEGF results in impaired capillary density and exacerbated heart failure (Shiojima et al., 2005; Oka et al., 2014; Yin et al., 2016).

Studies conducted to explore the effect of QSYQ on cardiac angiogenesis have been increasing. Results revealed that QSYQ could preserve microvascular function in patients undergoing elective percutaneous coronary intervention (He et al., 2017), improve coronary microcirculation in diabetic rats (Jin et al., 2010), and promote angiogenesis after myocardial infarction in rats (Zhang et al., 2010). All these studies suggested that QSYQ may exert a beneficial effect on cardiac angiogenesis. Hence, we hypothesized that QSYQ could prevent cardiac remodeling and thus improve cardiac dysfunction by ameliorating cardiac microvessel impairment in pressure overload-induced heart failure. Our study aimed to investigate the effects of QSYQ on heart failure induced by pressure overload and explore the underlying mechanism.

## Materials and methods

### Animals

Male C57BL/6 mice (8–10 weeks old, 20–25 g) were purchased from the Experimental Animal Center of Wuhan University (Wuhan, China). All these mice were raised in the animal center of Wuhan University of Science and Technology (Wuhan, China). After 1 week of adaptation period, the mice were randomly divided into four groups (*n* ≥ 6 per group): Sham + NS group, Sham + QSYQ group, TAC + NS group, and TAC + QSYQ group. Pressure overload was induced through transverse aortic constriction (TAC), as described previously (Takimoto et al., 2005). The mice were anesthetized and subjected to sham surgery or TAC with a 27-gauge blunted needle for 8 weeks. QSYQ (Tianjin Tasly Pharmaceutical Co., Ltd., Tianjin, China) dissolved in normal saline (NS) was administered intragastrically (3.5 mg/100 g/day) in the Sham + QSYQ and TAC + QSYQ groups. In the Sham + NS and TAC + NS groups, NS was provided every day intragastrically. All animal experimental protocols were approved by the Animal Care and Use Committee of Wuhan University of Science and Technology and were in accordance with the NIH Guide for the Care and Use of Laboratory Animals. For echocardiographic analysis, mice were anesthetized initially with 2% isoflurane, and then at 1% during the examination. Heart catheterization was performed under intraperitoneal pentobarbital anesthesia (30 mg/kg body weight). Then, all animals were sacrificed under deep anesthesia with excessive inhalation of isoflurane, and cardiac tissue samples were snap-frozen in liquid nitrogen for Western blots or real-time PCR or were fixed with 4% neutral formalin for paraffin embedding, and subsequent analysis were performed in a blind fashion. Additionally, in order to prevent personal, laboratory, and environmental exposure to potentially biohazards or harmful materials, all experimental protocols were in accordance with the guidance from the 5th edition of the Biosafety in Microbiological and Biomedical Laboratories and World Health Organization's Laboratory Biosecurity Guidance.

### Echocardiography

The mice were tied after anesthesia administration, and a pet razor was used to remove their chest hairs. Subsequently, echocardiographic analysis was performed using a high-resolution imaging system with a 30-MHz high-frequency scan head (VisualSonics Vevo1100, VisualSonics Inc., Toronto, Canada) at 8 weeks after TAC, as described previously (Wu et al., 2010). M-mode images of parasternal short axis at the tip of the papillary muscle level were recorded. Left ventricular internal dimension in diastole (LVIDd), left ventricular internal dimension in systole (LVIDs), left ventricular end-diastolic volume (LVEDV), left ventricular end-systolic volume (LVESV), ejection fraction (EF), and fractional shortening (FS) were recorded.

### Hemodynamics

Hemodynamic analysis was performed with the Millar pressure-volume system (AInstruments, New South Wales, Australia). The protocols were described previously (Yang et al., 2015). Briefly, the animals were tied after anesthesia administration. The right common carotid artery was separated, with the proximal artery clamped and the distal artery pulled with a suture. Subsequently, an incision of a suitable size was made and the catheter was inserted into the artery. Finally, the clamp was loosened and the catheter was pushed toward the left ventricular cavity. Maximal slope of systolic pressure increment (Max dP/dt) and the minimal slope of diastolic pressure decrement (Min dP/dt) were recorded for at least 15 min.

### Enzyme-linked immunosorbent assay (ELISA) analysis

After hemodynamic measurement, a blood sample was collected from the right carotid artery. Serum levels of VEGF was measured by the corresponding ELISA Kits (R&D System, Minneapolis, MN, USA), according to manufacturer's instruction. Absorbances were read at 450 nm (Microplate Reader Model 550, Bio-Rad, Hercules, CA, USA). All samples were assayed in triplicate.

### Immunohistochemical staining

After the mice were sacrificed, the hearts were separated, fixed with 4% neutral formalin, dehydrated, and prepared in paraffin sections. To evaluate cardiac fibrosis, Masson staining kit (Jiancheng Bioengineering Institute, Nanjing, China) was employed according to the manufacturer's instructions. For CD31 staining, sections were incubated with anti-CD31 antibody (1:100; ABclonal Biotech., MA), and a 0.3% hydrogen peroxide solution was used to block the endogenous peroxidase activity. Following application of an appropriate biotinylated secondary antibody, sections were developed with DAB substrates, and the number of vessels was determined with Image-Pro Plus 6. For TUNEL staining, TACS TdT *in situ* Apoptosis Detection Kit—DAB (R&D Systems, Minneapolis, MN) was employed. Tissue sections were visualized under light microscope (Nikon, Tokyo, Japan). For immunofluorescent staining, tissue sections were incubated with anti-actinin2 antibody (1:100; GeneTex, Inc.) overnight and with a secondary antibody for 90 min. DAPI solution was subsequently added for 5 min to stain the nuclei. Subsequently, sections were imaged under a Nikon DXM1200 fluorescence microscope with Nikon InfinityOptical System (Nikon, Tokyo, Japan).

The level of fibrosis were quantified in 5 microscopic fields chosen randomly at × 100 magnification under the microscope. Image analysis software (Image-Pro Plus 6.0) was used to calculate the ratio of myocardial collagen fiber area to all area and the average value was taken. Quantitative assessment of capillary density were identified by CD31 staining. Five microscopic fields were chosen randomly at × 400 magnification under the microscope. Image analysis software (Image-Pro Plus 6.0) was used to calculate the ratio of CD31 positive cell to all area, and the average value was taken. Similar to CD31 staining, quantitative assessment of cardiac apoptosis were quantified in 5 microscopic fields chosen randomly at × 400 magnification under the microscope. Image analysis software (Image-Pro Plus 6.0) was used to calculate the ratio of TUNEL positive cell to all area, and the average value was taken.

### Western blot analysis

Western blot analysis was performed as described previously (Wang et al., 2003). In brief, cardiac lysates were homogenized in an radio immunoprecipitation assay (RIPA) buffer with protease and phosphatase inhibitors cocktail (Roche). Total protein lysates were separated using SDS/PAGE, transferred overnight to a PVDF membrane, and immunoblotted. The membranes were soaked in a blocking buffer (5% BSA) for 1 h at room temperature and then incubated overnight at 4°C with primary antibodies. VEGF, Akt, serin473-phospho-Akt (p-Akt), endothelial NO synthase (eNOS), and serin1177-phospho-eNOS (p-eNOS) protein levels were performed using specific primary antibodies as belows: rabbit anti-VEGF (1:1000 dilution; abcam); rabbit anti-Akt (1:1000 dilution; Cell signaling); rabbit anti-p-Akt (1:2000 dilution; Cell signaling); rabbit anti-eNOS, (1:1500 dilution; Affinity); rabbit anti-p-eNOS, (1:1000 dilution; Affinity). After washing in TBST, membranes were incubated with the appropriate horseradish peroxidase-conjugated secondary antibodies at room temperature for 1 h. The protein signal detection was performed using the SuperSignal ECL system (ThermoScientific, Waltham, Massachusetts) and bands were analyzed by ImageJ software. Band intensity was normalized to that of glyceraldehyde-3-phosphate dehydrogenase (GAPDH). The p-Akt to t-Akt ratio and p-eNOS to t-eNOS ratio indicated, respectively, the levels of Akt and eNOS phosphorylation in the heart.

### Quantitative real-time RT-PCR (qRT-PCR)

Total RNAs were extracted from myocardial tissue samples with the Trizol reagent (Invitrogen, Carlsbad, CA) and reverse transcribed to cDNA using the reverse translation PCR kit (Thermo Fisher Scientific, Waltham, MA). Thereafter, the cDNA was used as a template, and specific primers as well as SYBR Green (Thermo Fisher Scientific, Waltham, MA) were used to detect the expression of the targets on a 7900HT Real-Time PCR System (Applied Biosystems, Foster City, CA). Reactions were performed in triplicate, the level of mRNA was normalized to that of GAPDH, and analysis was performed using the 2-ΔΔCt method.

The PCR primers used were as follows: VEGF forward, 5′-TCGTCCAACTTCTGGGCTCTT-3′; and reverse, 5′-CCCCTCTCCTCTTCCTTCTCT-3′; GAPDH forward, 5′ -ATGGGTGTGAACCACGAGA-3′; and reverse, 5′- CAGGGATGATGTTCTGGGCA-3′.

### Statistical analysis

All data were presented as mean ± standard error of the mean (SEM). Data were analyzed with SPSS (version 19.0; SPSS, Chicago, USA). Two-way analysis of variance was performed to compare multiple groups followed by Student-Newman-Keuls post hoc test. *P* < 0.05 was considered statistically significant.

## Results

### QSYQ protects against TAC-induced cardiac dysfunction in mice

**Figure 1:**
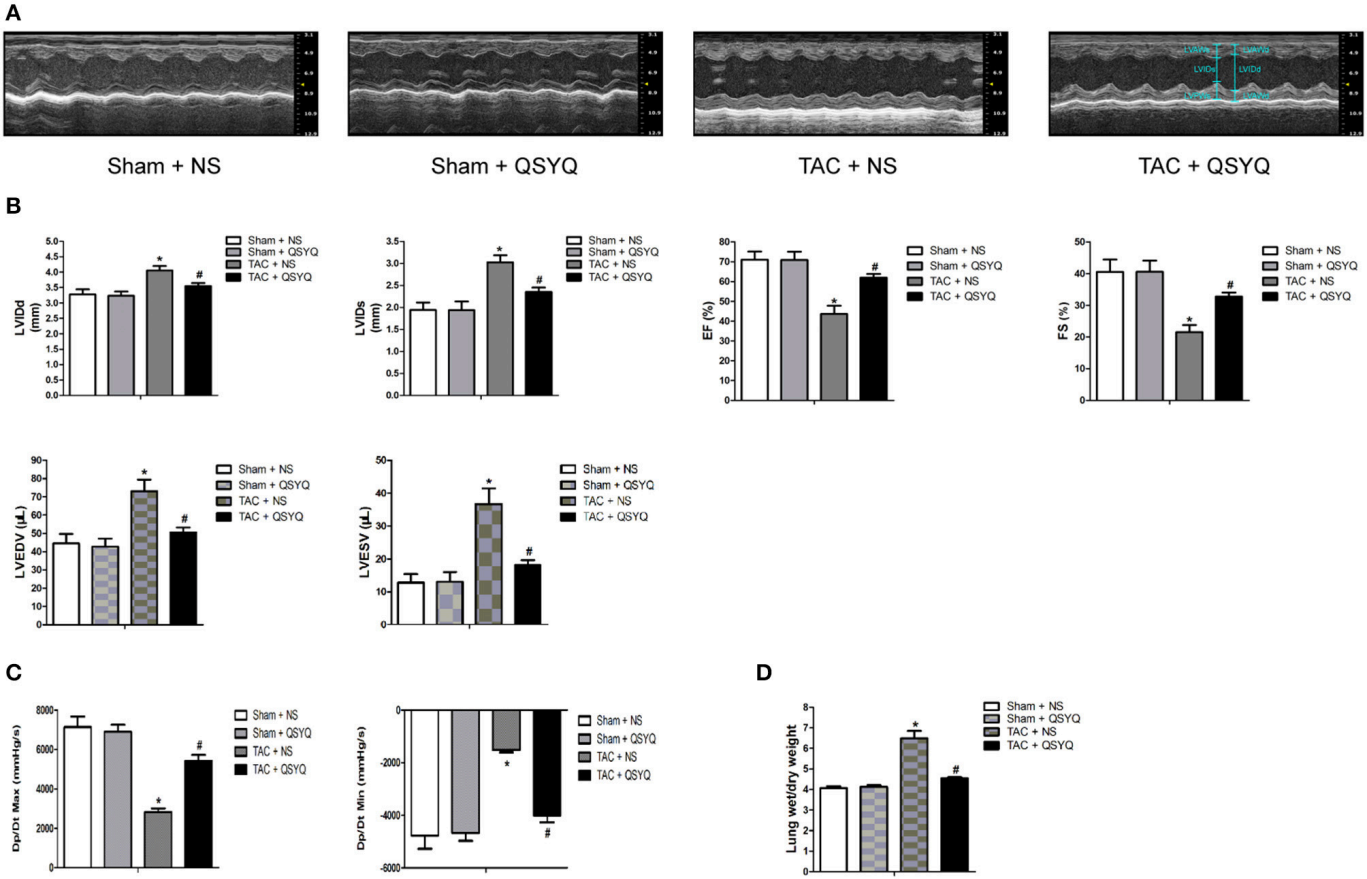
QSYQ improved cardiac function and hemodynamics of heart failure in TAC mice. **(A)** Representative M-mode echocardiography in the Sham + NS, Sham + QSYQ, TAC + NS, and TAC + QSYQ groups at 8 weeks after TAC, showing evidence of heart failure with LV dilatation in TAC mice; QSYQ prevented the development of cardiac failure in TAC mice. **(B)** Echocardiographic analysis of mice 8 weeks after TAC. **(C)** Hemodynamic analysis was performed with Millar cardiac catheter system in the four groups. **(D)** The lung wet/dry weight ratio. LVAWd, left ventricular anterior wall thickness in diastole; LVAWs, left ventricular anterior wall thickness in systole; LVPWd, left ventricular posterior wall thickness in diastole; LVPWs, left ventricular anterior wall thickness in systole; LVIDd, left ventricular internal dimension in diastole; LVIDs, left ventricular internal dimension in systole; EF, ejection fraction; FS, fractional shortening; LVEDV, left ventricular end-diastolic volume; LVESV, left ventricular end-systolic volume; Max dP/dt, maximal slope of systolic pressure increment; Min dP/dt, minimal slope of diastolic pressure decrement. Data shown are means ± SEM (*n* = 6–7 per group). *P < 0.05 vs. Sham + NS group; ^#^*P* < 0.05 vs. TAC + NS group.

To assess the cardiac function and chamber size in the mice, we used M-mode echocardiography in the Sham + NS, Sham + QSYQ, TAC + NS, and TAC + QSYQ groups 8 weeks after TAC. Figure 1A, which shows the representative M-mode echocardiography in the four groups 8 weeks after TAC, demonstrates that heart failure with LV dilatation existed in TAC mice and that QSYQ prevented the development of LV dilatation in TAC mice. Specifically, as shown in Figures 1B,D, EF and FS in the TAC + NS group significantly decreased compared with that in the Sham + NS group, which was accompanied by increased LVIDd and LVIDs (see Supplementary Material online, Table S1), as well as increased LVEDV, LVESV, and lung wet/dry weight ratio, thereby suggesting heart failure in the TAC + NS group. After treatment with QSYQ, EF, and FS significantly recovered, and LVIDd and LVIDs, as well as LVEDV, LVESV, and lung wet/dry weight ratio, also improved in the TAC + NS group.

To further evaluate the hemodynamics in the mice, Max dP/dt and Min dP/dt, which reflect LV systolic function and diastolic function, respectively, were determined with Millar cardiac catheter system. Figure 1C shows that compared with those in the Sham + NS group, Max dP/dt, and Min dP/dt in the TAC + NS group were significantly reduced, whereas in the TAC + QSYQ group, Max dP/dt, and Min dP/dt significantly improved (see Supplementary Material online, Table S2).

No significant difference in heart rate among the four groups was observed. Echocardiographic and hemodynamic examinations indicated that QSYQ markedly improved cardiac dysfunction in TAC mice.

### QSYQ improves loss of organization of myocardial fibers in TAC mice

**Figure 2:**
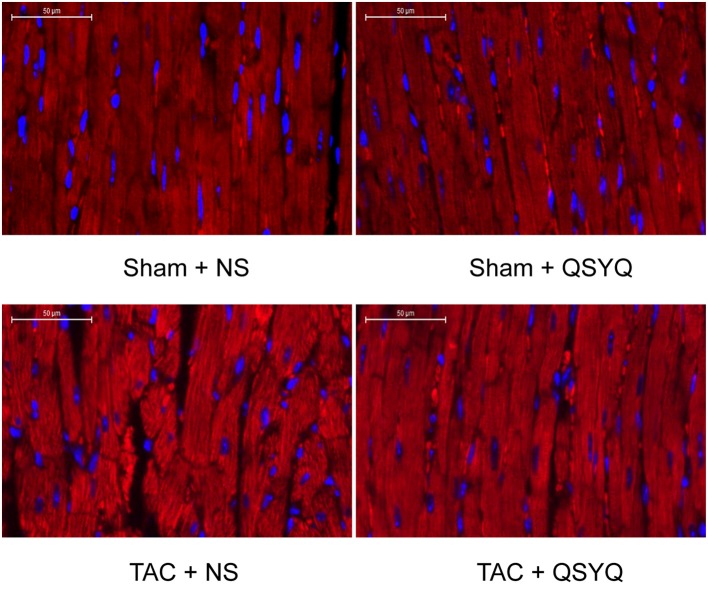
QSYQ improved loss of organization of myocardial fibers in TAC mice. Representative photographs of sections of left ventricles stained with anti-actinin2 antibody with a secondary antibody conjugated to Cy3. Sections were obtained from the Sham + NS, Sham + QSYQ, TAC + NS, and TAC + QSYQ groups 8 weeks after TAC. The Sham + NS group showed ordered myocardial fibers. By contrast, hearts from TAC mice showed disrupted myocardial fibers. QSYQ prevented loss of organization of myocardial fibers in TAC mice.

To further investigate the effect of QSYQ on the structure of myocardial tissues in TAC mice, immunofluorescent staining with anti-actinin2 antibody was performed to show the organization of the myocardial fibers. Figure 2 shows that the organization of the myocardial fibers in the Sham + NS group was ordered. By contrast, the TAC + NS group showed disrupted myocardial fibers. Myocardial fiber disruption in the TAC + QSYQ significantly improved compared with that in the TAC + NS group.

### QSYQ attenuates cardiac fibrosis in TAC mice

**Figure 3:**
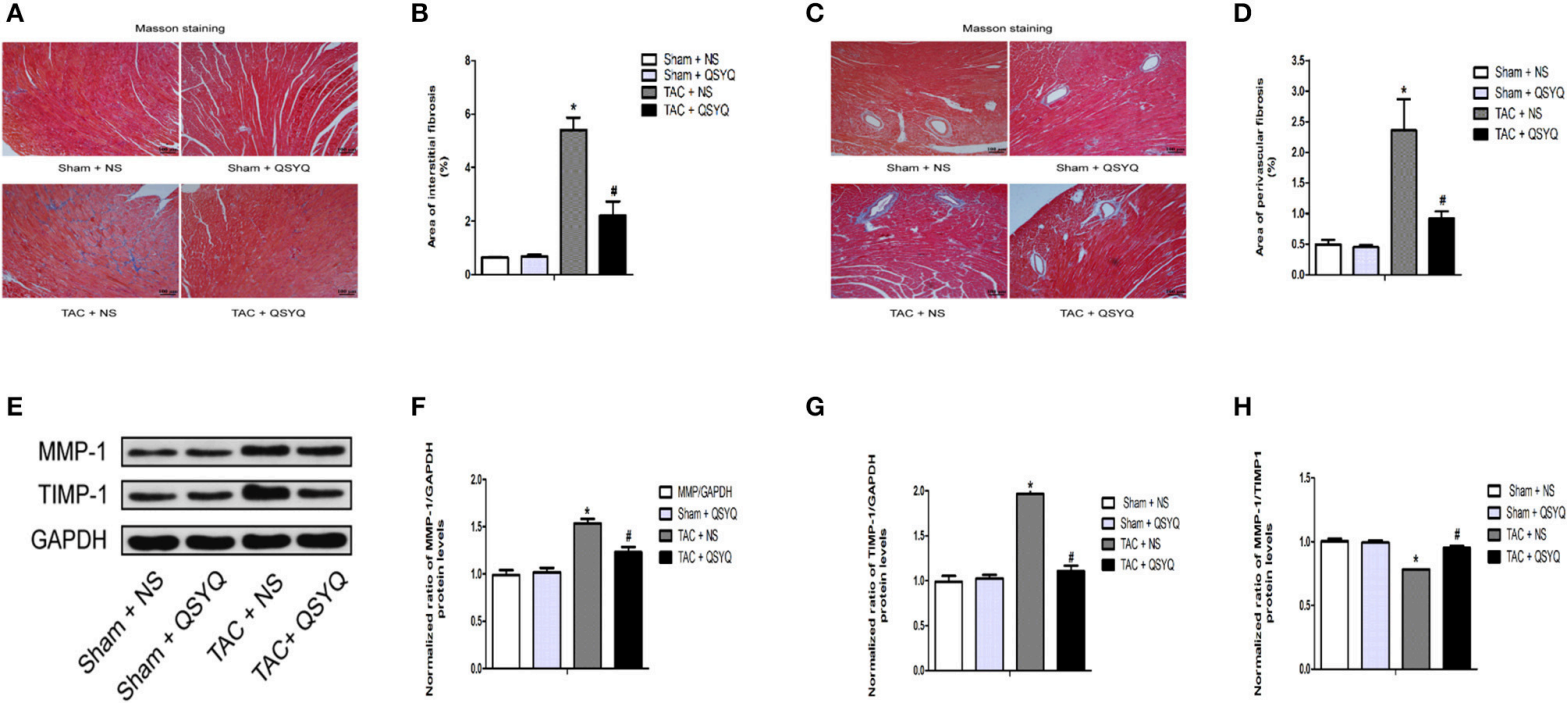
QSYQ attenuated cardiac fibrosis in TAC mice. **(A)** Representative photographs of sections of left ventricles with Masson trichrome staining in the Sham + NS, Sham + QSYQ, TAC + NS, and TAC + QSYQ groups 8 weeks after TAC. Blue staining indicates interstitial fibrosis. **(B)** Quantitative analysis of interstitial fibrosis in the four groups. **(C)** Representative photographs of sections of left ventricles with Masson trichrome staining in the four groups. Blue staining indicates perivascular fibrosis. **(D)** Quantitative analysis of perivascular fibrosis in the four groups. **(E)** Representative immunoblots for MMP-1 and TIMP-1 in the heart. **(F–H)** Quantitation of protein levels of MMP-1 and TIMP-1 in the heart. *P < 0.05 vs. Sham + NS group; ^#^*P* < 0.05 vs. TAC + NS group; data shown are means ± SEM (*n* = 6 per group).

Cardiac fibrosis, including myocardial interstitial fibrosis and perivascular fibrosis, is a vital indicator for the assessment of cardiac remodeling. To investigate whether QSYQ exerts effects on cardiac fibrosis, Masson trichrome staining in myocardial tissue was performed. Figures 3A,B shows that in the Sham + NS group, extremely few blue fibers exist, suggesting insignificant myocardial interstitial fibrosis, whereas the blue fiber in the TAC + NS group was significantly increased and distributed irregularly, suggesting that myocardial interstitial fibrosis was evident. After treatment with QSYQ, the blue fiber significantly decreased. Similarly, Figures 3C,D shows that no significant perivascular collagen deposition was observed in the Sham + NS group, whereas perivascular collagen deposition significantly increased in the TAC + NS group. Moreover, perivascular collagen deposition significantly decreased after treatment with QSYQ.

Besides, we investigated the effect of QSYQ on the regulation of MMP-1 and TIMP-1. Figures 3E–H showed that the protein expression of MMP-1 and TIMP-1 in heart was increased in TAC + NS group compared with sham + NS group, but the ratio of MMP-1/TIMP-1 was decreased. However, the QSYQ produced a significant decrease in the protein expression of MMP-1 and TIMP-1 in TAC mice and a significant increase in the ratio of MMP-1/TIMP-1. These results indicated that QSYQ can improve cardiac fiborsis in TAC mice by regulating the expression of MMP-1 and TIMP-1.

### QSYQ reduces cardiac apoptosis in TAC mice

**Figure 4:**
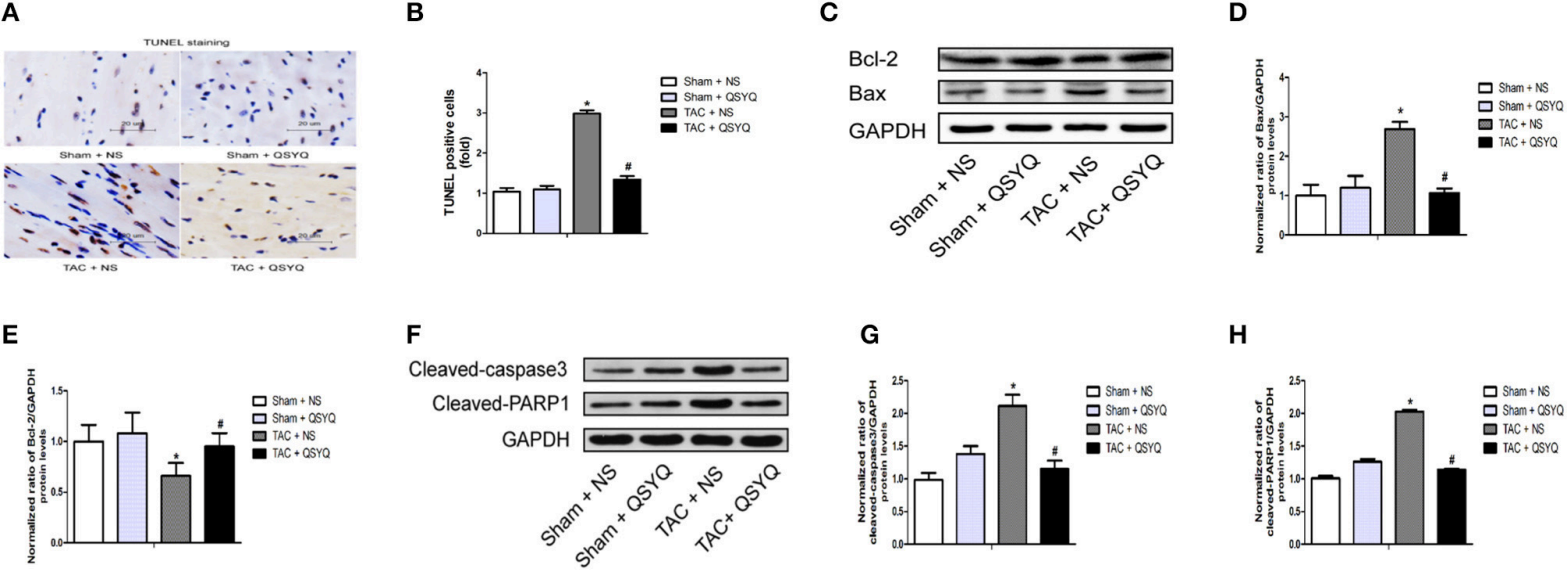
QSYQ reduced cardiac apoptosis in TAC mice. **(A)** Representative photographs of staining in heart sections. **(B)** Quantitative analysis of TUNEL staining in heart sections (*P < 0.05 vs. Sham + NS group; #*P* < 0.05 vs. TAC + NS group); data shown are means ± SEM (*n* = 6 per group). **(C)** Representative immunoblots for Bax and Bcl-2 in the heart. **(D–E)** Quantitation of protein levels of Bax and Bcl-2 in the heart. **(F)** Representative immunoblots for cleaved-caspase 3 and cleaved-PARP1 in the heart. **(G–H)** Quantitation of protein levels of cleaved-caspase 3 and cleaved-PARP1 in the heart. (*P < 0.05 vs. Sham + NS group; ^#^*P* < 0.05 vs. TAC + NS group); data shown are means ± SEM (*n* = 3 per group).

To evaluate the effect of QSYQ on cardiac apoptosis, TUNEL staining in heart sections was performed. Extremely few TUNEL-positive cells were observed in the Sham + NS group, whereas in the TAC + NS group, TUNEL-positive cells significantly increased, suggesting apparent cardiac apoptosis; however, a statistically significant decrease in TUNEL-positive cells was found after QSYQ treatment (Figures 4A,B). In addition, Western blot analysis was performed to evaluate the protein expression levels of Bax and Bcl-2. As shown in Figures 4C–E, the TAC + NS group had higher protein expression levels of Bax and lower protein expression levels of Bcl-2, which suggested elevated cardiac apoptosis. Conversely, compared with the TAC + NS group, the TAC + QSYQ group had remarkably decreased Bax expression levels and increased Bcl-2 expression levels, which suggested decreased cardiac apoptosis. Also, the other apoptosis-related protein expression levels of cleaved-caspase 3 and cleaved-PARP1 were examined. In line with the results of Bax, similar results were observed (Figures 4F–H). In addition, our results observed that no significant difference was found in the protein expression levels of p53 and FasL between sham + NS group and TAC + NS group and QSYQ has no significant effect on the protein expression levels of p53 and Fas ligand (FasL) in TAC mice (see Supplementary Material online, Figure S1).

### QSYQ alleviates cardiac microvessel impairment in TAC mice

**Figure 5:**
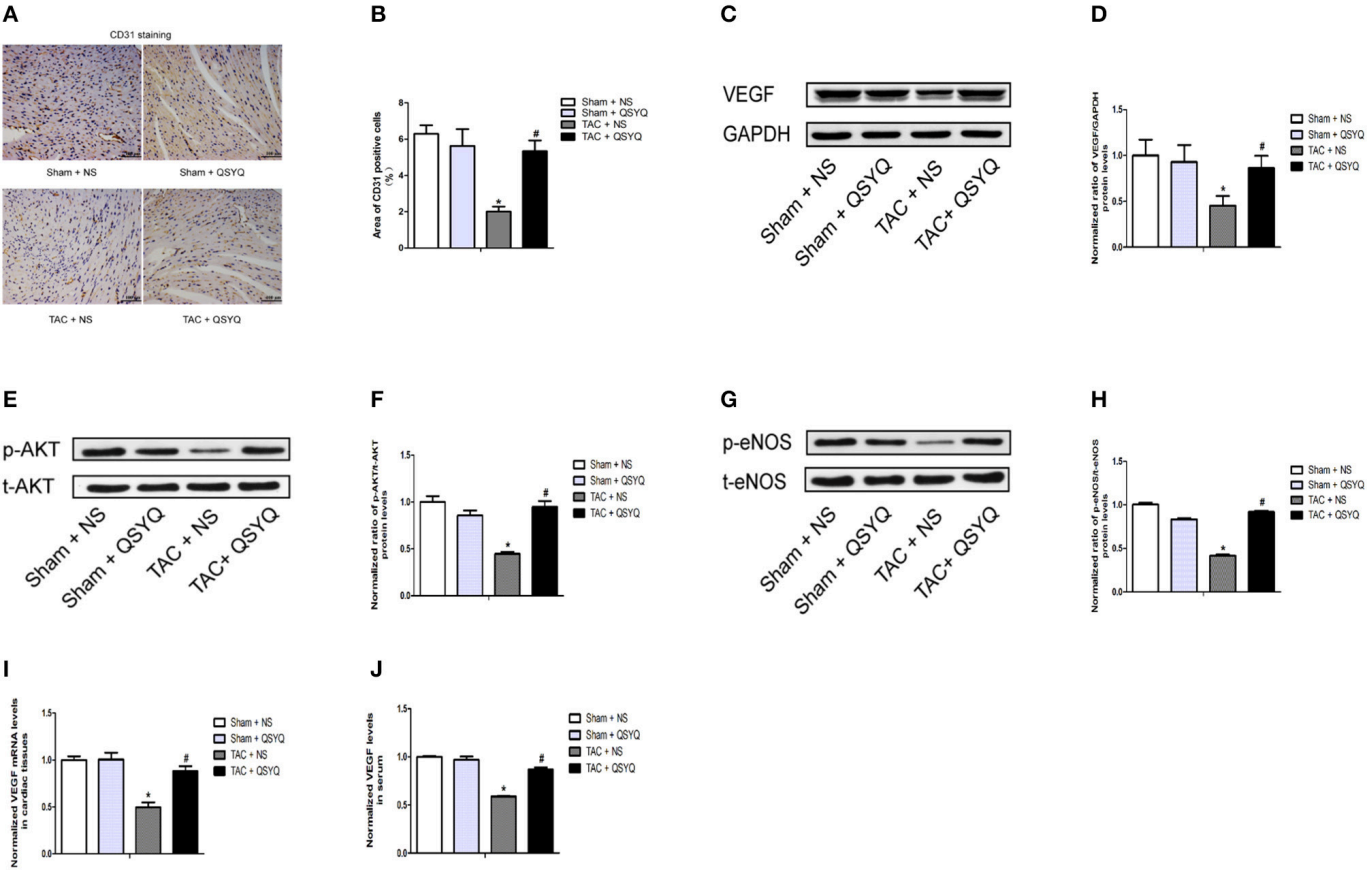
QSYQ alleviated cardiac microvessel impairment in TAC mice. **(A)** Representative photographs of sections of left ventricles with immunohistochemical staining with anti-CD31 antibody (scale bar, 100 μm). **(B)** Quantitation of CD31 expression level in the heart (*P < 0.05 vs. Sham + NS group; ^#^*P* < 0.05 vs. TAC + NS group); data shown are means ± SEM (*n* ≥ 4 per group). **(C)** Representative immunoblots for VEGF in the heart. **(D)** Quantitative analysis of cardiac expression of VEGF measured by western blot. **(E)** Representative immunoblots for t-Akt (total-Akt) and p-Akt (phospho-Akt) in the heart. **(F)** Quantitative analysis of cardiac expression of t-Akt and p-Akt measured by western blot. **(G)** Representative immunoblots for t-eNOS (total-eNOS) and p-eNOS (phospho-eNOS) in the heart. **(H)** Quantitative analysis of cardiac expression of t-eNOS and p-eNOS measured by western blot. **(I)** Quantitation of VEGF expression level in the heart measured by real-time PCR. **(J)** Quantitation of VEGF expression level in plasma measured by elisa. The p-Akt to t-Akt ratio and p-eNOS to t-eNOS ratio indicated, respectively, the levels of Akt and eNOS phosphorylation in the heart. *P < 0.05 vs. Sham + NS group; ^#^*P* < 0.05 vs. TAC + NS group; data shown are means ± SEM (*n* = 3 per group).

To explore the role of QSYQ in cardiac angiogenesis, immunohistochemical staining with anti-CD31 antibody was performed. As shown in Figures 5A,B, the expression of CD31, which is an indicator of tissue microvessel density, in the heart was significantly decreased in the TAC + NS group. In the TAC + QSYQ group, the CD31 expression level was remarkably elevated, suggesting that treatment with QSYQ reversed the reduction in microvessel density induced by TAC.

Western blots showed that the protein expression levels of VEGF in the TAC + NS group was significantly decreased compared with those in the Sham + NS group; treatment with QSYQ significantly increased VEGF expression levels in TAC mice (Figures 5C,D). Moreover, we found that VEGF mRNA expression level in the TAC + NS group was significantly decreased compared with that in the TAC + QSYQ group, whereas the VEGF mRNA expression level in the TAC + QSYQ group was significantly elevated, suggesting that treatment with QSYQ reversed the reduction in microvessel density induced by TAC (Figure 5I) (see Supplementary Material online, Figure S3). Similar results were determined in serum by ELISA (Figure 5J).

Interestingly, as shown in Figures 5E–H, we also found that a significant reduction of Akt activation and eNOS phosphorylation in TAC + NS group was observed compared with those in sham + NS group, which was reflected by the ratio of phospho-Akt/total-Akt (p-Akt/t-Akt) and phospho-eNOS/total-eNOS (p-eNOS/t-eNOS), respectively. Surprisingly, after treatment with QSYQ, the Akt activation and eNOS phosphorylation was significantly increased in TAC + NS group.

These results indicated that cardiac VEGF/Akt/eNOS pathway in the TAC + NS group was strongly suppressed compared with those in the Sham + NS group and the QSYQ was able to induce the activation of cardiac VEGF/Akt/eNOS pathway. In addition, our results observed that no significant difference was found in the expression of microRNA-223-3p (mir-223-3p) between sham + NS group and TAC + NS group and QSYQ has no significant effect on the expression of mir-223-3p in TAC mice (see Supplementary Material online, Figure S2).

## Discussion

The aim of this study was to evaluate the potential effects of QSYQ on TAC-induced heart failure in mice. Our results showed that treatment with QSYQ could protect against TAC-induced cardiac dysfunction and disrupted myocardial fiber structure. Moreover, treatment with QSYQ significantly improved cardiac apoptosis and cardiac fibrosis in TAC-induced heart failure, and we also observed that QSYQ could increase the expression levels of VEGF and promote cardiac angiogenesis.

To assess cardiac function, echocardiographic and hemodynamic examinations were performed, and immunofluorescent staining with anti-actinin2 antibody was employed to determine the organization of the myocardial fibers. Previous studies showed that QSYQ improves cardiac function in a rat model of myocardial ischemia induced by left anterior descending coronary artery ligation (JianXin et al., 2016). Additionally, QSYQ was also reported to effectively attenuate pressure overload-induced disorders in cardiac function in a rat model of heart failure (Chen et al., 2015). QSYQ was also found to ameliorate doxorubicin-induced myocardial structure damage and cardiac dysfunction in rats (Tang et al., 2013). Consistent with these reports, our study demonstrated that QSYQ could improve TAC-induced disorders in cardiac structure and function.

Furthermore, cardiac apoptosis and cardiac fibrosis were examined to evaluate cardiac remodeling in TAC-induced heart failure. Previous studies showed that after treatment with QSYQ, cardiac fibrosis in left ventricle hypertrophy induced by pressure overload through ascending aortic stenosis was attenuated (Li et al., 2012). Moreover, QSYQ protected against left ventricular remodeling induced by left anterior descending coronary artery ligation by attenuating the AngII–NADPH oxidase pathway (Li et al., 2014) or downregulating the RAAS pathway (Wang et al., 2015b). Additionally, QSYQ could effectively improve the cardiac function in adriamycin-induced cardiomyopathy, and the probable mechanism of action could be the inhibition of myocardial cell apoptosis (Tong et al., 2013). In our study, we found that QSYQ could remarkably reduce cardiac apoptosis in TAC mice. A previous study reported QSYQ was able to suppress cardiac cox-p53-FasL mediated apoptosis pathway in heart failure model induced by ligation of left anterior descending coronary artery (Wang et al., 2015a). However, our results observed that no significant difference was found in the protein expression levels of p53 and FasL between sham + NS group and TAC + NS group and QSYQ has no significant effect on the protein expression levels of p53 and FasL in TAC mice. This discrepancy may result from the difference of heart failure model. In addition, we also observed that QSYQ could not only ameliorate myocardial interstitial fibrosis but also improve perivascular fibrosis in TAC mice, which is also reported by only few studies. Previous studies showed that QSYQ can adjust the myocardial collagen metabolism in the abdominal aorta coarctation rat by regulating the expression of MMP-1 and TIMP-1 (Lv et al., 2015), which was also observed in our study.

Given that angiogenesis plays a vital role in the pathophysiology of heart failure (Semenza, 2014; Oka et al., 2016), we also investigated whether QSYQ could affect cardiac angiogenesis in TAC-induced heart failure. No compelling evidence from previous studies on whether QSYQ has any influence on cardiac angiogenesis in heart failure exists. Nevertheless, a previous study reported that QSYQ accelerates angiogenesis after myocardial infarction in rats (Zhang et al., 2010). In our study, we found that treatment with QSYQ significantly attenuates cardiac apoptosis and cardiac fibrosis in TAC-induced heart failure, which was accompanied by an upregulation of VEGF expression levels and maintenance of microvessel density in the heart. This in turn revealed that the potential mechanism of QSYQ in preventing cardiac remodeling may be closely related to improved cardiac angiogenesis. Meanwhile, we also found that a significant reduction of Akt activation and eNOS phosphorylation in TAC + NS group was observed compared with those in sham + NS group, which was reflected by the ratio of phospho-Akt/total-Akt (p-Akt/t-Akt) and phospho-eNOS/total-eNOS (p-eNOS/t-eNOS), respectively. Surprisingly, after treatment with QSYQ, the Akt activation and eNOS phosphorylation was significantly increased in TAC + NS group.

These results indicated that cardiac VEGF/Akt/eNOS pathway in the TAC + NS group was strongly suppressed compared with those in the Sham + NS group and the QSYQ was able to induce the activation of cardiac VEGF/Akt/eNOS pathway. This revealed that the potential mechanism of QSYQ in improving cardiac angiogenesis may be closely related to the activation of cardiac VEGF/Akt/eNOS pathway. Previous studies reported mir-223-3p has the most significant upregulation in ischemic cardiac microvascular endothelial cells (CMECs) compared with normal CMECs (Dai et al., 2014) and QSYQ promote ischemic cardiac angiogenesis by downregulating mir-223-3p expression in rats ischemic CMECs derived from rats myocardial infarction model (Dai et al., 2016). However, our results observed that no significant difference was found in the expression of mir-223-3p between sham + NS group and TAC + NS group and QSYQ has no significant effect on the expression of mir-223-3p in TAC mice. This discrepancy may result from the difference of model.

Our study comprehensively investigated the effects of QSYQ on TAC-induced heart failure. Moreover, our study simultaneously investigated the associated potential mechanism. However, evidence on the association between the cardioprotective effects of QSYQ on TAC-induced heart failure and cardiac angiogenesis, as well as evidence on the association between the proangiogenic effects of QSYQ on TAC-induced heart failure and cardiac VEGF/Akt/eNOS pathway, is extremely weak. Studies with more rigorous methodologies are warranted to draw a more convincing conclusion, and repeatable research findings are essential to make the conclusion solid.

In conclusion, our study suggested that QSYQ exerts a cardioprotective effect on heart failure induced by pressure overload, and the potential mechanism may be closely associated with the improvement in impaired cardiac angiogenesis by QSYQ.

## Author contributions

GR and HR conceived of and designed the experiments, performed the experiments, analyzed the data, and contributed to the writing of the manuscript; CZ conceived of and designed the research and performed the experiments; XZ and CX conceived of and designed the experiments; LW conceived and designed the experiments and contributed to the writing of the manuscript. All authors read and approved the final manuscript.

### Conflict of interest statement

The authors declare that the research was conducted in the absence of any commercial or financial relationships that could be construed as a potential conflict of interest.

## References

[B1] Ahmad T. Fiuzat M. Felker G. M. O'Connor C. (2012). Novel biomarkers in chronic heart failure. Nat. Rev. Cardiol. 9, 347–359. 10.1038/nrcardio.2012.37 22450126

[B2] Andrae J. Gallini R. Betsholtz C. (2008). Role of platelet-derived growth factors in physiology and medicine. Genes Dev. 22, 1276–1312. 10.1101/gad.1653708 18483217 PMC2732412

[B3] Chen Y. Y. Li Q. Pan C. S. Yan L. Fan J. Y. He K.. (2015). QiShenYiQi Pills, a compound in Chinese medicine, protects against pressure overload-induced cardiac hypertrophy through a multi-component and multi-target mode. Sci. Rep. 5:11802. 10.1038/srep11802 26136154 PMC4488877

[B4] Cohn J. N. Ferrari R. Sharpe N. (2000). Cardiac remodeling–concepts and clinical implications: a consensus paper from an international forum on cardiac remodeling. Behalf of an International Forum on Cardiac Remodeling. J. Am. Coll. Cardiol. 35, 569–582. 10.1016/S0735-1097(99)00630-0 10716457

[B5] Dai G. H. Liu N. Zhu J. W. Yao J. Yang C. Ma P. Z.. (2016). Qi-Shen-Yi-Qi dripping pills promote angiogenesis of ischemic cardiac microvascular endothelial cells by regulating microRNA-223-3p expression. Evid. Based Complement. Alternat. Med. 2016:5057328. 10.1155/2016/5057328 27057198 PMC4761670

[B6] Dai G. H. Ma P. Z. Song X. B. Liu N. Zhang T. Wu B. (2014). MicroRNA-223-3p inhibits the angiogenesis of ischemic cardiac microvascular endothelial cells via affecting RPS6KB1/hif-1a signal pathway. PLoS ONE 9:e108468. 10.1371/journal.pone.0108468 25313822 PMC4196764

[B7] Dallabrida S. M. Ismail N. S. Pravda E. A. Parodi E. M. Dickie R. Durand E. M.. (2008). Integrin binding angiopoietin-1 monomers reduce cardiac hypertrophy. Faseb. J. 22, 3010–3023. 10.1096/fj.07-100966 18502941 PMC2493452

[B8] Dobaczewski M. Chen W. Frangogiannis N. G. (2011). Transforming growth factor (TGF)-beta signaling in cardiac remodeling. J. Mol. Cell. Cardiol. 51, 600–606. 10.1016/j.yjmcc.2010.10.033 21059352 PMC3072437

[B9] He G. X. Xie J. Jiang H. Tan W. Xu B. (2017). Effects of Qishen Yiqi Dripping Pills () in reducing myocardial injury and preserving microvascular function in patients undergoing elective percutaneous coronar*y* intervention: a pilot randomized study. Chin. J. Integr. Med. [Epub ahead of print]. 10.1007/s11655-017-2955-1 28470563

[B10] Ho C. Y. Lopez B. Coelho-Filho O. R. Lakdawala N. K. Cirino A. L. Jarolim P.. (2010). Myocardial fibrosis as an early manifestation of hypertrophic cardiomyopathy. N. Engl. J. Med. 363, 552–563. 10.1056/NEJMoa1002659 20818890 PMC3049917

[B11] Hou J. Kang Y. J. (2012). Regression of pathological cardiac hypertrophy: signaling pathways and therapeutic targets. Pharmacol. Ther. 135, 337–354. 10.1016/j.pharmthera.2012.06.006 22750195 PMC3458709

[B12] Hou Y. Z. Wang S. Zhao Z. Q. Wang X. L. Li B. Soh S. B.. (2013). Clinical assessment of complementary treatment with Qishen Yiqi dripping pills on ischemic heart failure: study protocol for a randomized, double-blind, multicenter, placebo-controlled trial (CACT-IHF). Trials 14:138. 10.1186/1745-6215-14-138 23672353 PMC3680306

[B13] JianXin C. Xue X. ZhongFeng L. Kuo G. FeiLong Z. ZhiHong L.. (2016). Qishen Yiqi Drop Pill improves cardiac function after myocardial ischemia. Sci. Rep. 6:24383. 10.1038/srep24383 27075394 PMC4830957

[B14] Jin M. Liu H. D. Zhang Y. H. (2010). [Study on acting mechanism of shenqi yiqi drop pill for intervening irido-microangiopathy in diabetic rats]. Zhongguo Zhong Xi Yi Jie He Za Zhi. 30, 174–177. 20462047

[B15] Kardami E. Detillieux K. Ma X. Jiang Z. Santiago J. J. Jimenez S. K.. (2007). Fibroblast growth factor-2 and cardioprotection. Heart Fail. Rev. 12, 267–277. 10.1007/s10741-007-9027-0 17516168

[B16] Kirkpatrick J. N. St John Sutton M. (2012). Assessment of ventricular remodeling in heart failure clinical trials. Curr. Heart. Fail. Rep. 9, 328–336. 10.1007/s11897-012-0116-6 22983907

[B17] Li C. Wang Y. Qiu Q. Shi T. Wu Y. Han J.. (2014). Qishenyiqi protects ligation-induced left ventricular remodeling by attenuating inflammation and fibrosis via STAT3 and NF-kappaB signaling pathway. PLoS ONE 9:e104255. 10.1371/journal.pone.0104255 25122164 PMC4133204

[B18] Li Y. C. Liu Y. Y. Hu B. H. Chang X. Fan J. Y. Sun K.. (2012). Attenuating effect of post-treatment with QiShen YiQi Pills on myocardial fibrosis in rat cardiac hypertrophy. Clin. Hemorheol. Microcirc. 51, 177–191. 10.3233/CH-2011-1523 22240383

[B19] Lv S. Wu M. Li M. Wang Q. Wang X. Xu L.. (2015). Effect of QiShenYiQi Pill on myocardial collagen metabolism in rats with partial abdominal aortic coarctation. Evid. Based Complement. Alternat. Med. 2015:415068. 10.1155/2015/415068 25861361 PMC4377429

[B20] Oka T. Akazawa H. Naito A. T. Komuro I. (2014). Angiogenesis and cardiac hypertrophy: maintenance of cardiac function and causative roles in heart failure. Circ. Res. 114, 565–571. 10.1161/CIRCRESAHA.114.300507 24481846

[B21] Oka T. Morita H. Komuro I. (2016). Novel molecular mechanisms and regeneration therapy for heart failure. J. Mol. Cell. Cardiol. 92, 46–51. 10.1016/j.yjmcc.2016.01.028 26829118

[B22] Semenza G. L. (2014). Hypoxia-inducible factor 1 and cardiovascular disease. Annu. Rev. Physiol. 76, 39–56. 10.1146/annurev-physiol-021113-170322 23988176 PMC4696033

[B23] Shah A. M. Mann D. L. (2011). In search of new therapeutic targets and strategies for heart failure: recent advances in basic science. Lancet 378, 704–712. 10.1016/S0140-6736(11)60894-5 21856484 PMC3486638

[B24] Shiojima I. Sato K. Izumiya Y. Schiekofer S. Ito M. Liao R.. (2005). Disruption of coordinated cardiac hypertrophy and angiogenesis contributes to the transition to heart failure. J. Clin. Invest. 115, 2108–2118. 10.1172/JCI24682 16075055 PMC1180541

[B25] Takimoto E. Champion H. C. Li M. Belardi D. Ren S. Rodriguez E. R.. (2005). Chronic inhibition of cyclic GMP phosphodiesterase 5A prevents and reverses cardiac hypertrophy. Nat. Med. 11, 214–222. 10.1038/nm1175 15665834

[B26] Tang D. X. Zhao H. P. Pan C. S. Liu Y. Y. Wei X. H. Yang X. Y.. (2013). QiShenYiQi Pills, a compound chinese medicine, ameliorates doxorubicin-induced myocardial structure damage and cardiac dysfunction in rats. Evid. Based Complement. Alternat. Med. 2013:480597. 10.1155/2013/480597 23533487 PMC3600323

[B27] Tong J. Y. Xu Y. J. Bian Y. P. Shen X. B. Yan L. Zhu X. Y. (2013). Effect and mechanism of Qishen Yiqi Pills on adriamycin- induced cardiomyopathy in mice. Chin. J. Nat. Med. 11, 514–518. 10.3724/SP.J.1009.2013.00514 24359776

[B28] Wang H. Lin L. Jiang J. Wang Y. Lu Z. Y. Bradbury J. A.. (2003). Up-regulation of endothelial nitric-oxide synthase by endothelium-derived hyperpolarizing factor involves mitogen-activated protein kinase and protein kinase C signaling pathways. J. Pharmacol. Exp. Ther 307, 753–764. 10.1124/jpet.103.052787 12975498

[B29] Wang J. Li C. Cao Y. Wang Q. Lu L. Chang H.. (2015a). Mechanism of QSYQ on anti-apoptosis mediated by different subtypes of cyclooxygenase in AMI induced heart failure rats. BMC Complement. Altern. Med. 15, 352. 10.1186/s12906-015-0869-z 26445960 PMC4597456

[B30] Wang J. Lu L. Wang Y. Wu Y. Han J. Wang W.. (2015b). Qishenyiqi dropping pill attenuates myocardial fibrosis in rats by inhibiting RAAS-mediated arachidonic acid inflammation. J. Ethnopharmacol. 176, 375–384. 10.1016/j.jep.2015.11.023 26590099

[B31] Wang S. H. Mao J. Y. Hou Y. Z. Wang J. Y. Wang X. L. Li Z. J. (2013). [Routine western medicine treatment plus qishen yiqi dripping pill for treating patients with chronic heart failure: a systematic review of randomized control trials]. Zhongguo Zhong Xi Yi Jie He Za Zhi. 33, 1468–1475. 24483105

[B32] Wang Y. Lin W. Li C. Singhal S. Jain G. Zhu L.. (2017). Multipronged therapeutic effects of Chinese Herbal Medicine Qishenyiqi in the treatment of acute myocardial infarction. Front Pharmacol 8:98. 10.3389/fphar.2017.00098 28303103 PMC5332380

[B33] Wang Y. Zhao X. Gao X. Nie X. Yang Y. Fan X. (2011). Development of fluorescence imaging-based assay for screening cardioprotective compounds from medicinal plants. Anal. Chim. Acta 702, 87–94. 10.1016/j.aca.2011.06.020 21819864

[B34] Wu J. Bu L. Gong H. Jiang G. Li L. Ma H.. (2010). Effects of heart rate and anesthetic timing on high-resolution echocardiographic assessment under isoflurane anesthesia in mice. J. Ultrasound Med. 29, 1771–1778. 10.7863/jum.2010.29.12.1771 21098849

[B35] Yang L. Ni L. Duan Q. Wang X. Chen C. Chen S. (2015). CYP epoxygenase 2J2 prevents cardiac fibrosis by suppression of transmission of pro-inflammation from cardiomyocytes to macrophages. Prostaglandins Other Lipid Mediat. 116–117, 64–75. 10.1016/j.prostaglandins.2015.01.004 PMC5533100 25686540

[B36] Yin Z. Zhao Y. Li H. Yan M. Zhou L. Chen C.. (2016). miR-320a mediates doxorubicin-induced cardiotoxicity by targeting VEGF signal pathway. Aging 8, 192–207. 10.18632/aging.100876 26837315 PMC4761722

[B37] Zhang L. Wang Y. Yu L. Liu L. Qu H. Wang Y.. (2010). QI-SHEN-YI-QI accelerates angiogenesis after myocardial infarction in rats. Int. J. Cardiol. 143, 105–109. 10.1016/j.ijcard.2008.11.210 19203810

