# Supplementary material for: Cardioprotective Effects of QiShenYiQi Dripping Pills on Transverse Aortic Constriction-Induced Heart Failure in Mice
Source: Front Physiol. 2018 Apr 3;9:324. doi: 10.3389/fphys.2018.00324 (PMC5891926; doi:10.3389/fphys.2018.00324)

**Supplementary** **Figure Legends**

**Supplementary Table 1.** Echocardiographic characteristics of Sham + NS, Sham + QSYQ, TAC + NS, TAC + QSYQ groups. Values shown are means ± SEM (n = 6–7 per group). LVIDd, left ventricular internal dimension in diastole; LVIDs, left ventricular internal dimension in systole; EF, ejection fraction; FS, fractional shortening. *P < 0.05 vs. Sham + NS group; #P < 0.05 vs. TAC + NS group.

**Supplementary Table 2.** Table 2 Comparison of haemodynamic characteristics for Sham + NS, Sham + QSYQ, TAC + NS, TAC + QSYQ groups. Values shown are means ± SEM (n = 6–7 per group). Max dP/dt, maximal slope of systolic pressure increment; Min dP/dt, minimal slope of diastolic pressure decrement. *P < 0.05 vs. Sham + NS group; #P < 0.05 vs. TAC + NS group.

**Supplementary Figure 1.** QSYQ has no significant effect on the protein expression levels of of p53 and Fas ligand (FasL) in TAC mice. (A) Representative immunoblots for p53 and FasL in the heart. (B–C) Quantitation of protein levels of p53 and FasL in the heart. data shown are means ± SEM (n = 3 per group).

**Supplementary Figure 2.** QSYQ has no significant effect on the the expression of mir-223-3p in TAC mice. The expression of miR-21-3p was measured by quantitative real-time PCR according to the manufacturer’s protocol (Ribobio), and U6 small nuclear RNA was used as an internal normalized reference. Each reaction was performed in triplicate, and the results were analysed using 22DDCt method. (A) The expression of miR-21-3p in the heart. (B) The [real time PCR melting curve analysis](http://www.sogou.com/link?url=hedJjaC291Pl05MTlF1Zk2XH0kc1pIdi1J9581whAPUoEK1J-uWNQppQulN8Xmjk_9S_0Y09Acwh5nxpDb3WRg..) for miR-21-3p. (C) The [real time PCR melting curve analysis](http://www.sogou.com/link?url=hedJjaC291Pl05MTlF1Zk2XH0kc1pIdi1J9581whAPUoEK1J-uWNQppQulN8Xmjk_9S_0Y09Acwh5nxpDb3WRg..) for U6 small nuclear RNA.

**Supplementary Figure 3.** A specific melting peak in [real time PCR melting curve analysis](http://www.sogou.com/link?url=hedJjaC291Pl05MTlF1Zk2XH0kc1pIdi1J9581whAPUoEK1J-uWNQppQulN8Xmjk_9S_0Y09Acwh5nxpDb3WRg..) for the GAPDH used primers in our study, which revealed the GAPDH used primers in our study is specific.

**Supplementary Table 1**

|  | **Sham + NS** | **Sham + QSYQ** | **TAC + NS** | **TAC + QSYQ** |
| --- | --- | --- | --- | --- |
| **Echocardiography** |  |  |  |  |
| Heart rate, bpm | 428±22 | 440±21 | 452±31 | 438±27 |
| LVIDd, mm | 3.28±0.16 | 3.23±0.14 | 4.05±0.14***** | 3.55±0.09**#** |
| LVIDs, mm | 1.94±0.17 | 1.94±0.19 | 3.03±0.16***** | 2.35±0.1**#** |
| EF, % | 71±4.1 | 70.9±4.1 | 43.6±4.2***** | 62±1.8**#** |
| FS, % | 40.5±3.9 | 40.6±3.5 | 21.5±2.3***** | 32.8±1.3**#** |
|  |  |  |  |  |

**Supplementary Table 2**

|  | **Sham + NS** | **Sham + QSYQ** | **TAC + NS** | **TAC + QSYQ** |
| --- | --- | --- | --- | --- |
| **Hemodynamics** |  |  |  |  |
| Heart rate, bpm | 475±22.8 | 501±15.1 | 473±21.9 | 486±18.8 |
| Max dP/dt, mmHg/s | 7144±530 | 6913±350 | 2831±183***** | 5426±313**#** |
| Min dP/dt, mmHg/s | -4764±506 | -4665±305 | -1514±94***** | -3999±264**#** |

**Supplementary** **Figure 1**


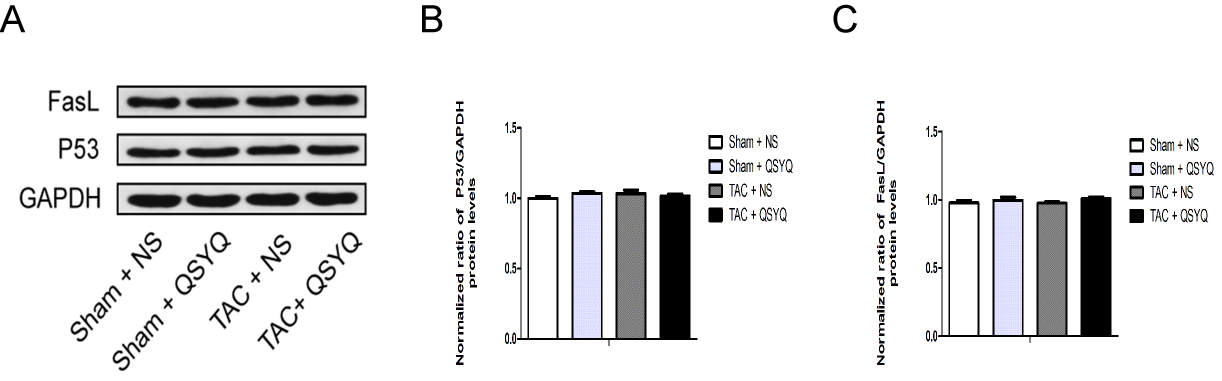


**Supplementary** **Figure 2**


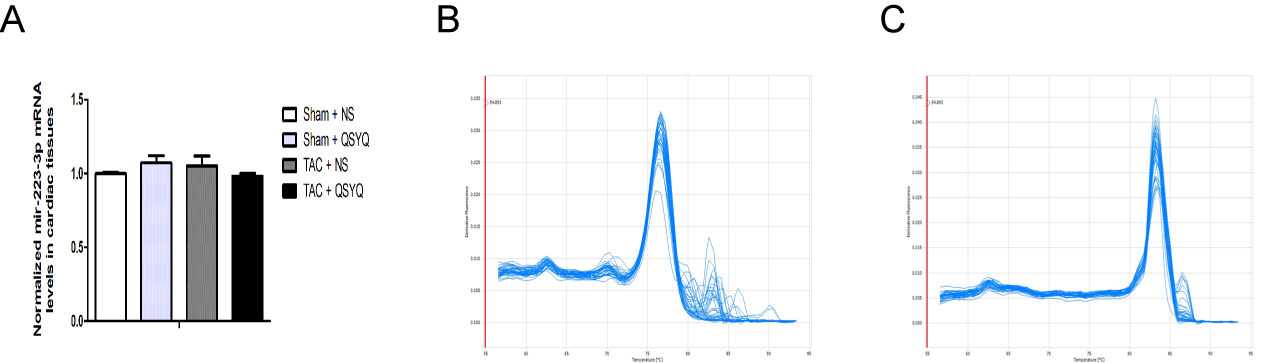


**Supplementary** **Figure 3**


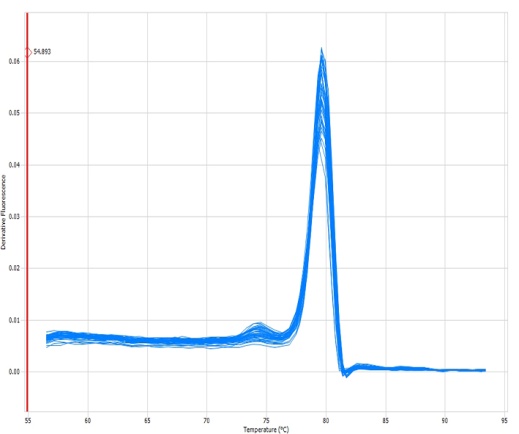

Supplement: Supplementary file 1 [file DataSheet1.docx]
